# Pathological, microscopic, and molecular diagnosis of paratuberculosis/John’s disease in naturally infected dromedary camel (*Camelus dromedarius*)

**DOI:** 10.14202/vetworld.2023.1277-1283

**Published:** 2023-06-08

**Authors:** El Tigani Ahmed El Tigani-Asil, Ghada El Derdiri Abdelwahab, El Hadi Ahmed Mohamed Abdu, Abdelnasir Mohammed Adam Terab, Nasareldien Altaib Hussein Khalil, Zhaya Jaber Mohammed Al Marri, Mohd Farouk Yuosf, Asma Abdi Mohamed Shah, Abdelmalik Ibrahim Khalafalla, Hassan Zackaria Ali Ishag

**Affiliations:** 1 Biosecurity Affairs Division, Development and Innovation Sector, Abu Dhabi Agriculture and Food Safety Authority, Abu Dhabi, United Arab Emirates; 2 Extension Services and Animal Health Division, Animal Wealth Sector, Abu Dhabi Agriculture and Food Safety Authority, Abu Dhabi, United Arab Emirates

**Keywords:** acid-fast bacteria, dromedary camel, *Mycobacterium*, paratuberculosis

## Abstract

**Background and Aim:**

Paratuberculosis (PTB) or John’s disease is a chronic disease of ruminants impeding the reproduction and productivity of the livestock sector worldwide. Since there is a lack of pathological studies explaining the nature and development of the disease in camels, this study aimed to highlight the anatomopathological changes of PTB in camels, which may help in verifying and validating some diagnostic tests used to detect the etiology of the disease in camel tissues.

**Materials and Methods:**

In August 2017, at Alselaa border’s Veterinary Clinic of Al Dhafra Region, Western Abu Dhabi, UAE, one imported culled she-camel of 2 years old was subjected to clinical, microscopic, and anatomopathological investigations along with real-time quantitative polymerase chain reaction (q-PCR) to confirm the infection and correlate between clinical signs and pathological lesions of the PTB in dromedary camels.

**Results:**

Clinically, typical clinical signs compliant with the pathognomonic gross and histologic lesions of PTB were seen in naturally infected dromedary camel. As presumptive diagnosis microscopically, acid-fast coccobacillus bacterium clumps were demonstrated in direct fecal smears as well as in scraped mucosal and crushed mesenteric lymph node films, and in histopathological sections prepared from a necropsied animal and stained by Ziehl-Neelsen stain. Free and intracellular acid-fast clump phagosomes were further confirmed as *Mycobacterium avium* subsp. paratuberculosis by q-PCR.

**Conclusion:**

Clinical signs and pathological lesions of paratuberculosis in a dromedary camel were found to be similar to those of the other susceptible hosts.

## Introduction

Paratuberculosis (PTB) or John’s disease (JD) is a chronic, debilitating, and contagious disease of ruminants characterized by long-term persistent watery diarrhea, generalized weakness, and severe emaciation that inevitably ends in death [1–6]. Paratuberculosis is caused by *Mycobacterium avium* subsp. paratuberculosis (MAP), which is an environmental resistant pathogen that spreads worldwide and principally affects the health of a wide range of domestic and wild ruminants, carnivores, equines, birds, and camelids [7–11]. Wild animals, including ruminants, carnivores, equines, pigs, and birds, are susceptible to infection and may act as PTB reservoirs or infectious hosts can disseminate the pathogen to other susceptible hosts and obstacles the control and combatting of the disease [1, 3, 6, 12–14].

Based on phenotypic, genotypic, and host relationships, MAP is principally classified into two major groups, the sheep-type (also named S-type or Type I and III sub-lineages) and the cattle-type (also called C-type or Type-II sub-lineage) of which a bison strain (B-strain) is subgrouping [10, 15–17].

Although PTB has not yet been classified as a zoonotic disease, the bacterium MAP is implicated in the cause of fatal human inflammatory enteric disease known as Crohn’s disease, which is resembled the clinical form of PTB in animals and this reinforces its possible role as a zoonotic pathogen [18–21]. The disease has a significant global economic burden on the agricultural sector due to decreased productivity, waste of body weight, treatment costs, culling and compensation policies, and deaths [22, 23].

Dromedary camels are also reported to be infected by MAP which induces clinical and subclinical forms of the disease [24–27]. In Saudi Arabia, a typical case of MAP investigated by gross and microscopic pictures, has been detected from ileum and mesenteric lymph nodes taken from dromedary camels [28, 29]. MAP is transmitted orally by contaminated feed in adults or infected milk in sucking animals of clinical or subclinical infectious mothers [2].

The PTB is characterized by a long incubation period; hence, clinical signs of the disease usually appear in adult infected animals; however, the disease can occur in animals at any age over 1–2 years [6]. Clinical symptoms of PTB are attributed to enteropathy and chronic proliferative enteritis leading to long-term intermitted or progressive watery diarrhea responsible for wasting of the body condition and other nutrients. Finally, the disease ends by cachexia and inevitable death [30]. Subclinical cases constantly excrete bacteria in their feces, which are hazardous to susceptible animals and are thought to pose an epidemiological concern, particularly in an intensive animal production system [2, 7].

The pathological picture of clinical cases of PTB in true hosts is related to chronic, slowly proliferative, or granulomatous enteritis results in diffuse thickening of the mucosa, which is folded into transverse rugae, the crests of which may be congested, and the ileocecal and mesenteric lymph nodes and lymphatic vessels are enlarged and edematous, caseated, or calcified foci are sometimes found in the intestines and associated lymph nodes of sheep, goats, and cervid [30].

According to the severity and extension of infection, histological manifestation of PTB in animals is classified into focal, multifocal, and/or diffuse granulomatous inflammation [31]. Other secondary pathological lesions such as cachexia, dehydration, edema, anemia, and pica are attributed to nutrient deficiencies following prolonged diarrhea. Gross lesions are usually obscure in subclinical carries. Pathological changes in camelids are reported to be similar to those in cattle, with some differences regarding lymph node necrosis and mineralization [30].

Diagnosis of PTB is based on observing characteristic clinical symptoms correlated to pathognomonic gross or histologic lesions, microscopic demonstration of acid-fast bacterium, culturing, and polymerase chain reaction (PCR) confirmation [4, 32]. Cytopathological Ziehl-Neelsen staining technique of smears prepared from fecal, ileocecum mucosal scrappy, and mesenteric lymph node crush smears remains a golden diagnostic test for demonstration of free clumps or phagosomes of acid-fast MAP in the infected animal tissues [6]. Culturing and PCR procedures are more likely recommended for diagnosing subclinical cases of PTB due to the small number of microbes shed in the fecal material. Hence, propagation is necessary for identification [26, 33]. Genome-wide analysis of MAP was further performed in *Camelus dromedarius* in Saudi Arabia and was observed the circulation of MAP between sheep and camel shared the environment [34].

Controlling the disease is challenging and ineffective due to subclinical characteristics, high fecal shedding results in the spreading of the disease among susceptible herds, vertical transmission, late diagnosis, and lack of awareness [4, 14, 35].

To date, detailed information about MAP infection in camels is generally scanty, while early and timely diagnosis and identification of clinical and subclinical high-shedding animals are essential for PTB control and minimization of economic losses [36].

Therefore, this study aimed to provide a comprehensive diagnostic approach to the disease in camels using several techniques, including clinical observations, conventional microscopic, anatomopathological, and molecular biology techniques, which confirmed the incrimination of the MAP in the development of the disease in camel.

## Materials and Methods

### Ethical approval

Ethical approval was obtained from the research ethics committee, Abu Dhabi Agriculture and Food Safety Authority (ADAFSA-EA-03-2019). In addition, written consent (included in the sample request form, approved by the ADAFSA research ethics committee) for the use of samples and animals was obtained from the Camel’s owners before inclusion in the study.

### Study period and location

The study was conducted in August 2017 at Alselaa Boarder’s Veterinary Clinic in Al Dhafra Region, Abu Dhabi, UAE, for routine diagnosis

### Clinical examination

A female dromedary camel of around 2 years old with a history of chronic watery diarrhea, good appetite, pica, and emaciation was admitted. The case was clinically diagnosed as PTB and the necessary samples were collected for further tentative diagnosis using direct microscopy of acid-fast bacilli using Ziehl-Neelsen-staining method [37]. The culled she-camel was subjected to further anatomopathological and real-time quantitative PCR (q-PCR) investigations to confirm the infection and correlate between clinical signs and pathological lesions of the PTB in dromedary camel [38].

### Necropsy examination

Culled infected dromedary camel was subjected to postmortem examination [38, 39]. Gross pathological changes were described, and tissue samples from pathognomonic lesions were collected and fixed in 10% neutral buffered formal for histopathological analysis [40]. Crushed and mucosal scrapped smears from mesenteric lymph nodes and ileocecal mucosa were prepared for microscopic examination of acid-fast bacteria using the Ziehl-Neelsen staining technique [37]. Mesenteric lymph node and ileum specimens were also collected and preserved at –20°C for further molecular confirmatory tests.

### Microscopic examination

The Ziehl-Neelsen method of Acid-fast bacilli staining procedure was used to demonstrate pathogenic acid-fast bacteria involved in fecal samples and granulomatous animal lesions [2].

### Histopathological analysis

Intestine, mesenteric lymph node, and liver tissue samples were fixed in 10% neutral buffered formalin. Samples were then embedded in paraffin and cut into 5-μm thick serial sections. Sections were stained with Hematoxylin and Eosin (H&E) according to the method previously described by Redi [40] and images were acquired to examine the histopathological changes. A series of 5-μm thick sections of intestine and mesenteric lymph node were stained by a Ziehl-Neelsen staining kit according to manufacturer instructions (International Biomedical Supplies, Canada).

### Detection of the bacterial genome by q-PCR

The genomic DNA of the bacteria was extracted from ileum and lymph node tissues with QIAamp^®^ DNA Mini Kit (Qiagen, Hilden, Germany) following the manufacturer’s instructions. The bacterial genome was detected by q-PCR with the following primers and probe: F2: 5’ AATGACGGTTACGGAGGTGGT 3’, R2: 5’ GCAGTAATGGTCGGCCTTACC 3’ and probe: P2 5’ TCCAC GCCCG CCCAG ACAGG 3’ [41]. Light Cycler^®^ TaqMan Master Detection kit (Roche) was used for the PCR reaction, and the PCR mixture consisted of 8 μL of water, 1 μL of each primer (10 pmol), 1 μL of TaqMan probe, 4 μL of Master Mix (5×), and 5 μL of the DNA template. Amplification was performed on the LightCycler 2.0 Instrument (Roche, Life Science system) as per the previously described conditions [41]. Reactions were performed in duplicates. A non-template control sample was included in the run.

## Results and Discussion

**Figure-1:**
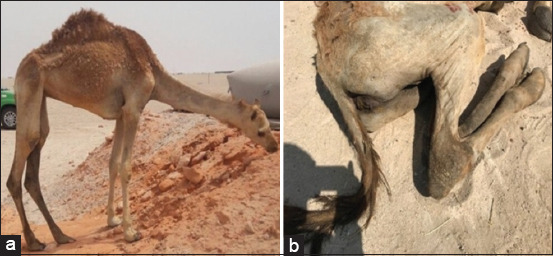
Clinical sings of paratuberculosis infected dromedary camel show (a): Emaciation, rough coat and sign of deviated appetite (pica) due to loss of rare nutrients (b): Evidence of diarrhea material stained the hind limbs.

Clinical examination of the infected dromedary camel showed typical clinical signs of PTB that included prolonged intermitted progressive watery diarrhea, severe emaciation, dehydration, edematous swelling of the ventral body sites, and deprivation of appetite (pica), possibly due to deficiency of some rare nutrients or gastrointestinal parasite infestation (Figure-1). The symptoms observed in this study appear to be similar to those described for MAP in camels and other animals [24, 25, 27, 42, 43]. In addition, the disease has been observed in a 2-year-old case, consistent with a previous study that described the infection of camels with MAP [28]. Other reports also indicated that the incidence of the disease is high in camels aged 2–5 years and that death occurs within 2–20 weeks of clinical illness [44]. However, other animals can be infected at any age above 1–2 years [6].

**Figure-2:**
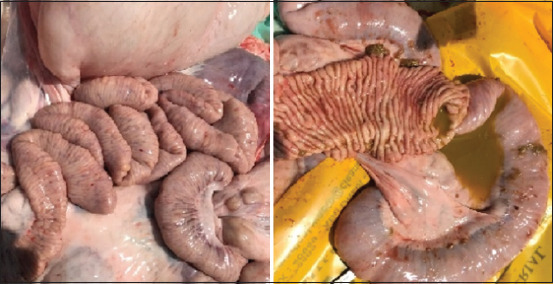
Dromedary camel ileocecal part and cut surface show remarkable diffuse edematous thickening, intestinal folding, and corrugations with some nodular lesions and watery ingesta.

**Figure-3:**
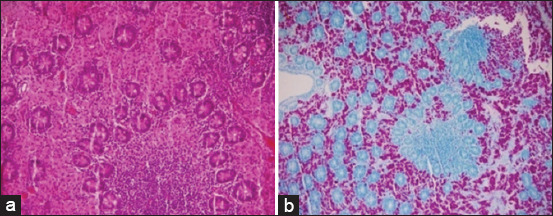
Intestine sections: (a) shows granulomatous enteritis evident on the lamina propria with proliferative mucoid glands (hematoxylin and eosin stain, 20×). (b) Clusters and clumps of acid-fast bacilli in the lamina propria and mucosa (Ziehl-Neelsen, 20×).

At necropsy, pathognomonic lesions of PTB were registered. The ileocecal part showed thickening and corrugations attributed to chronic proliferative enteritis. The cut surface of the ileum also revealed folding and corrugation of the mucosal surface (Figure-2). Histopathological sectioning of formalin-fixed intestinal tissues showed prominent proliferative enteritis characterized by stunted villi, lepromatous granuloma characterized by diffuse infiltration of closely packed macrophages and epithelioid cells together with lymphocytes in the mucosa and lamina propria resulting in considerable widening or thickening of the intestinal mucosa. Many mucous glands in the lamina propria suffered variable degrees of atrophy and disintegration (Figure-3a). A serial section of intestine tissue was stained by Ziehl-Neelsen, clusters of acid-fast bacilli in the lamina propria and mucosa were observed (Figure-3b).

**Figure-4:**
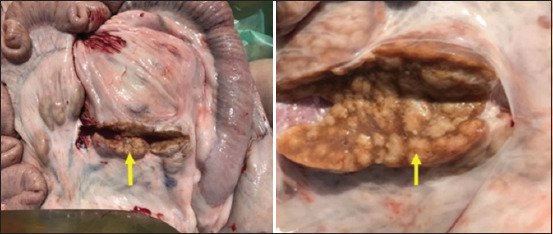
Mesenteric lymph nodes cross section shows remarkable swelling, edematous, nodular, and mottled appearance.

**Figure-5:**
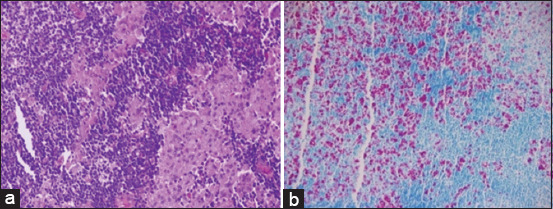
(a) Mesenteric lymph node section shows diffuse lepromatous granulomatous aggregation of macrophages, lymphocytes, plasma, and epithelioid cells. Giant cells were poorly noticed (hematoxylin and eosin stain, 20×): (b) Mesenteric lymph node: clusters of acid-fast bacilli (Ziehl-Neelsen, 200×).

**Figure-6:**
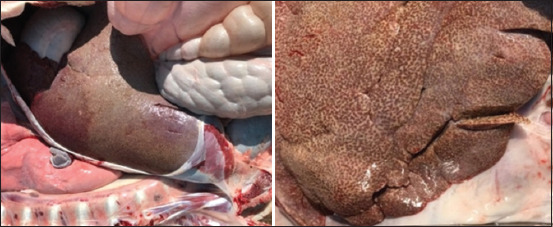
The liver was firm in consistency with diffuse mottling and grossly enhanced lobular pattern (accentuation of hepatic lobulation) giving the liver nutmeg appearance. The cut surface was also mottled with accentuation of hepatic lobules. This appearance is indicative of chronic passive congestion of the liver with hepatocellular degeneration and necrosis of the centrilobular hepatocytes as secondary sequelae of chronic enteritis.

**Figure-7:**
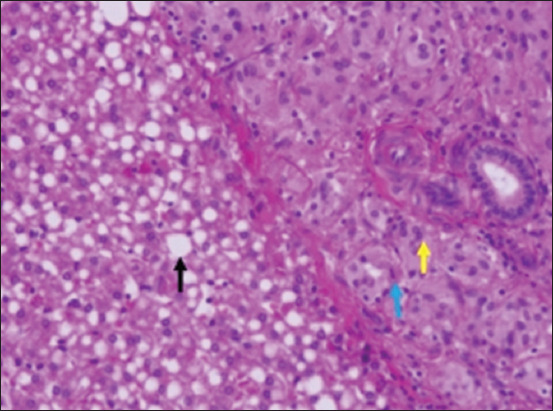
Liver section shows diffuse fatty change of hepatocytes (black arrows) in the centrilobular area. Hepatocytes in the portal area show signs of anaplasia structure particularly mitosis (blue arrow), pleomorphism with foaming appearance (yellow arrows) resembled to focal hepatic carcinoma.

**Figure-8:**
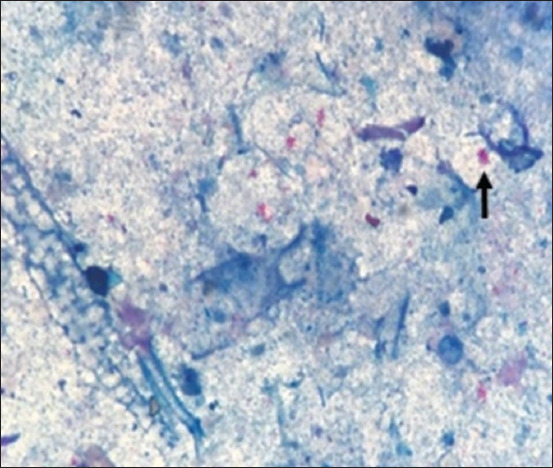
Fecal smear stained with Ziehl-Neelsen stain shows clumps of acid-fast coccobacilli bacteria; *Mycobacterium avium* subsp. paratuberculosis (arrow).

**Figure-9:**
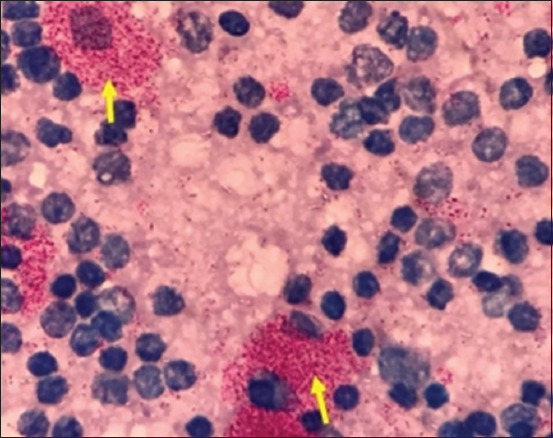
Mesenteric lymph nodes crush smear stained with Ziehl-Neelsen stain shows numerous closely packed acid-fast bacilli (*Mycobacterium avium* subsp. paratuberculosis) inside the cytoplasm of macrophages and epithelioid cells (arrows).

**Figure-10:**
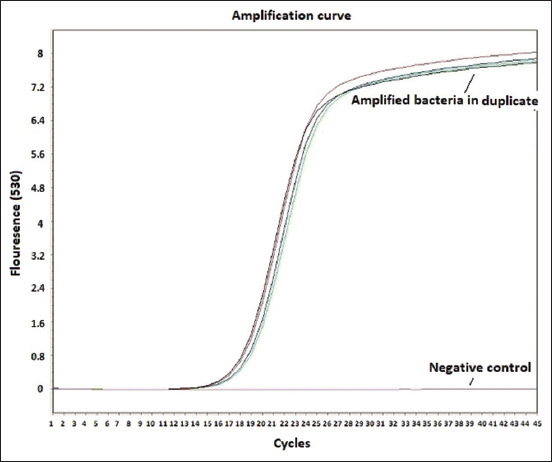
The amplification curve of specific *Mycobacterium avium* subsp. paratuberculosis from ileum and lymph node tissues. The control negative with straight line (no amplification) was also shown.

Remarkable enlargement of the mesenteric lymph nodes exhibited firm consistency, mottling appearance, and caseation was noticed (Figure-4). Mesenteric lymph nodes sections indicated a diffuse lepromatous reaction with a proliferation of macrophages and epithelioid cells mixed with lymphocytes and other mononuclear cells without evidence of caseation and calcification. Giant cells were poorly noticed (Figure-5a). Clumps of acid-fast coccobacilli bacteria were seen in the copy section of the mesenteric lymph node (Figure-5b). Other secondary pathological changes such as hepatic fatty degeneration, nutmeg liver due to passive congestion, hepatic necrosis, fatty change, and atrophy were also observed (Figures-6 and 7). Clinical profile of naturally PTB-infected dromedary camel reported here typically resembled the pathology of PTB in other ruminants [2, 45, 46]. Microscopic examination of smears prepared from the fecal sample of the infected case confirmed the shedding of acid-fast bacteria in the feces (Figure-8). As well, scraping smears from mesenteric lymph nodes and ileocecal mucosa stained with Ziehl-Neelsen stain showed numerous closely packed acid-fast phagosomes of MAP lodged inside the cytoplasm of macrophages and epithelioid cells (Figure-9). Formalin-fixed sections from mesenteric lymph nodes and ileum also demonstrated acid-fast clumps, consistent with previous findings [37]. Furthermore, we performed molecular analysis (q-PCR) with the bacteria targeting the IS900 gene. Our q-PCR results confirmed the presence of the bacteria in the samples with low Ct values (16.9–17.5) for both lymph node and intestine samples, as shown in Figure-10. Our results agree with previous reports that observed Ct values ranging from 17 to 32 for IS900 analysis of MAP [41]. The results of our study suggest that dromedary camels are susceptible to PTB pathogen, which induces severe pathological changes with prominent clinical presentation.

## Conclusion

Combining histopathological analysis with previously standard methods (clinical observation, Ziehl-Nielsen staining, and q-PCR) is very helpful in screening camels with suspected JD. Furthermore, Ziehl-Neelsen staining of a 5-μm thick section of the intestine and mesenteric lymph node could be applied in routine camel MAP diagnosis and demonstrate the infection’s severity. Analysis of the whole-genome sequence of the isolated pathogen is required to understand the evolution and adaptation nature of the pathogen in camels.

## Author’s Contributions

EAE: Conceptualization, methodology, data curation, visualization, and writing-original draft. GEA: Methodology and revised the manuscript. EAAE: Necropsy examination. AMAT: Methodology, data curation, visualization, and writing-original draft. NAHK: Revised the manuscript. ZJMA: Performed the histopathology. MFY: Performed the q-PCR analysis). AMAS: Conceptualization, funding, supervision, and project administration, AIK: Conceptualization and reviewed the manuscript. HZAI: Conceptualization, analysis, and interpretation and reviewed the manuscript. All authors have read, reviewed, and approved the final manuscript.
